# Relative risk of using odds ratios

**DOI:** 10.3325/cmj.2019.60.181

**Published:** 2019-04

**Authors:** Pero Hrabač, Vladimir Trkulja

**Affiliations:** 1 Department of Medical Statistics, Epidemiology, and Medical Informatics, “Andrija Štampar” School of Public Health, University of Zagreb School of Medicine, Zagreb, Croatia *phrabac@hiim.hr*; 2 Department of Pharmacology, Zagreb University School of Medicine, Zagreb, Croatia

In the previous issue, we addressed the concept of odds and of odds ratio as a measure of association between two binary variables. To exemplify it, we asked the hypothetical question – “Is there an association between the exposure to pharmacological estrogens and endometrial cancer (EC)?” – and outlined two hypothetical observational studies that could provide an answer. In respect to odds ratio, we focused on the first study, a cross-sectional sample of 738 women examined at a gynecology ward: 74 EC patients and 664 women without EC. In the first subset, 56 women (75.7%) had been previously exposed to estrogens, while in the latter subset 247 (41.3%) had been exposed to estrogens. The EC patients could be also designated as cases (“cases” of EC) and women without it could be designated as controls (or, “non-cases”), and the study could be termed a *case-control* study, with data summarized as in Table 1.

**Table 1:** Summary of data from a hypothetical study assessing the association between estrogen exposure and diagnosis of endometrial cancer

	Exposed to estrogens		
Carcinoma	yes	no	Total
yes	(a) 56	(b) 18	(a + b) 74
no	(c) 274	(d) 390	(c + d) 664
Total	(a + c) 330	(b + d) 408	738

We pointed out that the association between the two variables (EC, exposure to estrogens) in this study could be quantified based on prevalence and using 3 different measures:

1. *Prevalence difference*: 75.7% –41.3% = 34.4% (34.4% higher prevalence of a history of estrogen exposure among cases than among controls, in absolute terms)

2. *Prevalence ratio*: 75.7%/41.3% = 1.83 (83% higher prevalence among cases, in relative terms).

Numerically, two measures would differ if Table 1 was observed from a “different angle”, ie, if one was to look at the prevalence of EC among women with a history of exposure to estrogens (56/330, 17.0%) and without such a history (18/408, 4.4%), giving an absolute difference of 12.6% and a ratio of 3.86 (relative difference of 386%).

3. *Odds ratio*: which is 4.42 regardless of the way of “looking” at Table 1, ie, [(56/74)/(18/74)] / [(274/664)/(390/664)] = 4.42 and [(56/330)/(274/330)] / [(18/408)/(390/408)] = 4.42.

All measures suggested an association between estrogen exposure and EC, ie, a “tendency” of estrogen exposure and the fact of being diagnosed with EC to “group together.”

What we could not do with such a study was to detect the *risk* of developing EC if exposed to (pharmacological) estrogens. As we pointed out in the previous issue, strictly speaking, the risk can be estimated only based on newly established cases of EC, ie, prospectively, based on the incidence of EC among women exposed or not exposed to estrogens who meet the basic condition that at the time of the start of exposure they are all free of EC. In this respect, we outlined a second (hypothetical) study involving a total of 738 women, all free of EC at the beginning of the observation, 330 of whom were then (for some reason) exposed to estrogens and 408 who were not. Such a study could be designated as a prospective cohort study, and its subsets could be designated as the exposed cohort (n = 330) and non-exposed or control cohort (n = 408). Over a subsequent 5-year period, EC was diagnosed in 56/330 exposed women (incidence or risk 17.0%) and in 18/408 non-exposed women (incidence or risk 4.4%). Obviously, data could again be summarized as in Table 1, but there is only “one way of looking at the table” (incidence of EC in the exposed vs incidence in the non-exposed), and the association between the two variables (a presumed risk factor and the outcome) could be quantified using any of the 3 measures:

1. *Absolute difference in the risk of EC between exposed and non-exposed or absolute risk difference* (ARD): 17.0%-4.4% = 12.6%

2. *Relative difference in the risk of EC between exposed and non*-*exposed or risk ratio or relative risk (RR)*: 17.0%/4.4% = 3.85

3. *Odds ratio (OR), as the odds of EC among exposed/odds of EC among non-exposed*: [(56/330)/(274/330)] / [(18/408)/(390/408)] = 4.42

**Note**. (i) ARD and RR are numerically identical as “one version” of prevalence difference and prevalence ratio (see above), but their *conceptual* meaning is different; (ii) OR is one of the possible association measures in this study but OR *does not quantify a difference in risk*, ie, OR *is not* relative risk, hence, based on OR, one should not conclude that “the risk of EC is 4.42 higher in exposed than in non-exposed women” – the risk is absolutely 12.6% higher and relatively 3.85 times higher. On the other hand, the odds are 4.42 times higher; (iii) numerically, OR and RR differ. It is only when the incidence of events (outcomes) is relatively low (ie, up to 10% at the most) or extremely high (ie, >90%) that OR and RR are numerically close.

With clearly more intuitive and simpler measures of association, like prevalence difference or ratio and absolute risk difference or risk ratio, why would one want to use OR as a measure of (strength) of association? The point is that in observational studies (eg, case-control and prospective cohort studies), there are many other potential factors that could be associated with or influence the occurrence (or presence) of EC, for example, age, family history of EC, parity, endocrine diseases, etc. When assessing the association between the exposure to pharmacological estrogens and EC, one actually needs to try to assess the existence of an *independent* association, ie, an association under the conditions in which all other potentially relevant factors in the exposed and non-exposed (or cases and controls) women are “identical.” This, of course, is practically impossible to achieve physically, but is attempted in statistical models that need to try to account for all these factors (*confounders*), hence there is a need to *model* the outcome measures. Both OR and RR have a *skewed* distribution, and in order to be appropriate for modeling they need to be corrected, which is achieved by logarithmic transformation. Historically, methods to model log(odds) were developed before the methods to model log(risk). Therefore, OR is still commonly used as a measure of association in case-control studies (but prevalence ratio can also be modeled) and prospective cohort studies (although relative risk, ie, risk ratio, can also be modeled).

